# Stress and Wellbeing during the COVID-19 Pandemic: A Mixed-Methods Exploration of Frontline Homelessness Services Staff Experiences in Scotland

**DOI:** 10.3390/ijerph19063659

**Published:** 2022-03-19

**Authors:** Hannah Carver, Tracey Price, Danilo Falzon, Peter McCulloch, Tessa Parkes

**Affiliations:** 1 Salvation Army Centre for Addiction Services and Research, Faculty of Social Sciences, University of Stirling, Stirling FK9 4LA, UK; tracey.price@stir.ac.uk (T.P.); d.c.falzon@stir.ac.uk (D.F.); t.s.parkes@stir.ac.uk (T.P.); 2 School of Health Sciences, University of Dundee, Dundee DD1 4HJ, UK; p.y.mcculloch@dundee.ac.uk

**Keywords:** homelessness, wellbeing, staff, organisational culture, burnout, Scotland, occupational stress, COVID-19, Maslach Burnout Inventory

## Abstract

Staff working in homelessness services often find the work rewarding yet challenging, and the sector experiences high levels of staff burnout and staff turnover. During the COVID-19 pandemic, staff working in these services faced particularly stressful working conditions. This study explored the experiences of stress and wellbeing among those working in frontline homelessness service roles during the early stages of the pandemic in Scotland. Semi-structured interviews were conducted with 18 participants, 11 of whom completed the Maslach Burnout Inventory (MBI). Qualitative data were analysed using Framework Analysis in NVivo, informed by the Revised Transactional Model of occupational stress and coping. MBI data were analysed using descriptive statistics. The COVID-19 pandemic positively and negatively impacted participants’ lives and roles. Organisational culture acted as a magnifying glass for pre-pandemic practices: for some, the pandemic brought teams and staff closer together, creating a better working environment. For others, it led to fragmentation and frustration. Participants discussed coping strategies and recommendations for the future to protect staff wellbeing. Quantitative data suggested that participants were not experiencing burnout, although some were at heightened risk. Future research should explore the longer-term impact of the pandemic on homelessness service staff outcomes.

## 1. Introduction

### 1.1. Overview of the Literature

Homelessness refers to situations where individuals or families do not have access to suitable, stable or permanent housing. This includes people who are sleeping rough, in temporary or insecure housing arrangements, people in residential treatment centres, and those who live temporarily with family or friends [1]. There are multiple reasons why a person could become homeless but childhood trauma, institutional care or relationship breakdowns are common reasons [2]. Relatedly, many people experience problems with mental health, physical health and/or substance use [3]. A range of homelessness services exist in Scotland to provide a combination of accommodation and support. These include hostels, outreach and housing support.

Working in the homelessness sector can be challenging for many reasons. Establishing relationships with people with acute, multiple, and complex needs can be difficult, requiring time, patience and empathy [4]. Homelessness workers often feel that they do not have enough time to meet the needs of their clients and keep up with the external pressures they face when attempting to secure housing and wider social and health services for their clients [5]. Staff also face the additional stress of working at a time when deaths due to homelessness, drugs and alcohol remain consistently high [6,7,8]. The wellbeing of staff in frontline care roles, such as in the homelessness sector, can also influence the standard of care provided [9]. Staff often have to balance their own wellbeing with the challenges of supporting clients [10]. While there is no universal definition of wellbeing, in this study, we use the following definition: “the combination of feeling good and functioning effectively” [11,12]. We chose this definition as it is sufficiently broad and fits with the concepts relevant in this study, for example having control over one’s life, a sense of purpose, and experiencing happiness [12]. People facing homelessness are some of the most marginalised people in society [13], so the factors that support staff wellbeing in times of stress and strain must be better understood in order to ensure that clients are provided with suitable care and support.

Many people in frontline caring roles are driven by a strong desire to make a difference, which can act as a strong motivator for their work [14]. Evidence of having made a difference can lead to staff feeling enthusiastic, emotionally invested, and committed to the work, regardless of the challenges faced in the role or the sector itself [15]. Attending to the needs of clients can, however, be time consuming, and the broader system of services can be challenging to navigate [16]. This can lead to frustration for staff, and role conflict and ambiguity due to working in a complex system, which is part of a climate of increasing demand and reduced resources [17]. People who enter the homelessness system have often faced stigma when trying to access services, which means that they may distrust the efforts of staff and find engaging in support difficult [18]. Delays and challenges in the staff–client relationship can also lead to staff feeling disillusioned with their roles. Over time, particularly if support and supervision are insufficient, staff can begin to feel as though they are not good enough [9]. Such experiences can be detrimental to staff wellbeing and lead to a gradual build-up of stress and strain [19].

Organisational and individual personal factors can contribute to staff wellbeing. Organisational factors include management support, positive team dynamics and reflective practices [20,21]. Reflective practice refers to formal or informal arrangements that support staff to reflect honestly and openly on the emotional components of their work [22,23]. These practices can take place within teams, with managers via supervision, or with external professionals. Individual factors such as personality traits, support networks, and preferred coping strategies can also influence staff resilience in managing the potential stress involved in the role [24]. Psychological resilience is described as the ability to bounce back from adverse experiences, adapting and maintaining a sense of wellbeing [25]. Social capital, the support networks and resources someone has available to cope with difficulties [26], may also be an important factor within homelessness services [16].

Despite the positive strategies that can be deployed to maintain staff wellbeing, a large body of literature points to the risk of staff burnout within the homelessness sector [19,27,28,29]. The concept of burnout is complex and the term is often used interchangeably with compassion fatigue, vicarious trauma and secondary traumatic stress [30]. Although there are links between these concepts, each has notable differences. Our focus in this paper will be on the concept of burnout. Maslach et al. (2001) suggest that burnout has three main dimensions: an overwhelming sense of exhaustion; feeling unable to manage; and feeling emotionally detached and cynical [19]. Burnout can have an impact on physical and mental health, with symptoms including persistent ill health, somatic symptoms, and poor mental health outcomes. Staff burnout contributes to high absenteeism and high turnover within the homelessness sector [31]. Burnout can occur in any role where there is a high workload, role conflict or ambiguity, and/or a feeling of being unsupported. It is not necessarily linked to working with people who have experienced trauma, or who face challenging life situations [30].

While working in a frontline homelessness role is already recognised as being potentially stressful, the COVID-19 pandemic created an additional, “formidable challenge” [32]. The COVID-19 pandemic was declared globally in March 2020 [33]. Governments across the globe began to impose lockdown restrictions to reduce transmission of the disease, with the first case in Scotland being announced on 11 March 2020, and a nationwide lockdown starting on 24 March 2020 [34]. In many countries, including Scotland, people were only permitted to leave their homes for essential purposes such as obtaining food supplies and medicine [35,36]. Frontline care work, medical roles, and maintaining critical infrastructures such as utilities and food were deemed essential, and those holding these roles were asked to continue working as usual. On 31 March 2020, the Scottish Government published information for homelessness services [34], which included guidance around social distancing, use of personal protective equipment (PPE), ventilation, and hygiene. Frontline homelessness services also had to adapt quickly to provide shelter for those who did not have suitable accommodation in order to prevent transmission of the disease amongst a group who were highly vulnerable [28]. Across many high-income countries (e.g., UK, US, Canada, and Australia), vacant hotels were converted into emergency hostels. For many, the new homelessness hotels enabled co-location of services to provide essential care such as access to medication, mental health support and drug and/or alcohol harm reduction [37]. However, the layering effect of pre-existing stress within the sector, and the need for rapid change, provided a potential threat to staff wellbeing.

Disaster response and rapid change dominated the homelessness sector in many countries in the early months of the pandemic. Numerous international studies have been conducted to examine the wellbeing of frontline health care workers during the pandemic in a range of countries (e.g., [24,38,39]), but virtually no research has focused on frontline homelessness workers. Kerman et al. (2021) surveyed 201 frontline homelessness workers in Canada before and during the pandemic and found that for 79.5%, their mental health had worsened [32]. Many were working more hours during the pandemic, and, despite this, a high proportion were experiencing financial difficulty, compounding the experience of stress. The study also noted that 41% met the criteria for post-traumatic stress disorder and reported increased exposure to traumatic situations during the pandemic. While many studies indicate that organisational factors such as supervision and reflective practices can reduce the likelihood of worker burnout, the Kerman et al. study found that the working environment had little impact on stress outcomes during the pandemic [32]. Other research has highlighted the impact of COVID-19 on the homelessness sector in Canada, with safety concerns, increased workload, and lack of training and PPE being discussed [40]. However, no studies examining the experiences of homelessness workers during the COVID-19 pandemic have been conducted outside of Canada. The aim of this study was to address this gap by exploring the interplay of organisational factors and personal coping strategies for frontline homelessness workers during the early stages of the COVID-19 pandemic in Scotland.

### 1.2. Theoretical Approach

Over the last five decades, multiple models have been proposed to better understand the factors that contribute to stress, strain, and coping among those in frontline caring roles [41,42,43,44]. Our study drew on the Goh et al. (2010) Revised Transactional Model (RTM) of occupational stress and coping to understand study participant experiences [42]. In the RTM, stressful events (stressors) are not necessarily one-off instances of stress but the cumulative effect of working in a potentially high-stress environment. Goh et al. (2010) argue that self-appraisals of stress, strain, and coping are ongoing in a high stress working environment. As new events occur, there are constant feedback loops, where individuals consider the event, coping resources, and the extent to which the stress presents a threat to wellbeing [42]. The RTM proposes that when an event is perceived as threatening or stressful, an individual will consider the available coping resources they have to deal with the event. When stress levels are raised, emotional and/or problem-focused coping strategies are activated. If these coping strategies fail to resolve the stressor, then stress levels will increase when individuals experience another stressful event [42]. In the context of this study, the compounding of potential stressors (with the pre-existing strain being a primary stressor and the pandemic a secondary stressor) offers an opportunity to gain insights into coping strategies and organisational factors to support staff emotional, psychological, physical and mental wellbeing during times of stress.

### 1.3. Study Aim

The aim of this mixed-methods study was to examine the experiences of frontline homelessness services staff, the strategies used to support coping, and their need for support and supervision, in order to provide recommendations for the sector. This study addressed the following research questions:

1. What challenges do frontline homelessness services staff in Scotland face in terms of stress, wellbeing, burnout and mental health during COVID-19?
2. How are staff coping, and what are their support needs?
3. What lessons can be learned for the homelessness sector in Scotland and beyond?

## 2. Materials and Methods

### 2.1. Study Design and Ethics

A mixed-methods approach was used, involving semi-structured interviews and completion of the Maslach Burnout Inventory (MBI). Ethical approval was granted by University of Stirling’s General University Ethics Panel (GUEP; paper 903) and the Ethics Subgroup of the Research Coordinating Council of The Salvation Army (RCC-EAN200505). All data collection was completed remotely due to COVID-19 restrictions.

Potential participants were identified by creating a list of all third sector (not-for-profit) homelessness service providers in Scotland and emailing service managers with information about this study, asking them to suggest staff members who might be willing to participate and met the study inclusion criteria: those currently working in frontline roles (i.e., not management). Service managers passed on information about this study to their teams (by email, at team meetings, and individually) and were asked to either provide contact details for interested staff or ask staff to contact the research team directly. Word of mouth and Twitter were also used to recruit participants directly, with interested organisations/individuals asked to contact H.C., who then passed the information to one of the researchers (T.Pr., D.F. or P.M.).

### 2.2. Interviews: Recruitment, Process and Analysis

Semi-structured interviews were conducted by three researchers (T.P. (Tracey Price), D.F. and P.M.) during the period June–October 2020. Participants were contacted by email and invited to take part in either a telephone or online interview. The initial email provided a participant information sheet which detailed the purpose and process of this study. Written informed consent was granted prior to each interview. All interviews were audio recorded with permission and lasted an average of 60 min. The interview schedule (Supplementary File S1) covered participants’ experiences of stress and wellbeing prior to and during the pandemic, as well as their coping strategies and support received, and recommendations for the future. After each interview, participants were given a debrief sheet, providing further information about this study and support available. Researchers captured their experiences and reflections in fieldnotes which supplemented data analysis, by informing the coding framework.

The interviews were transcribed in full and analysed thematically using Framework Analysis [45], informed by the RTM. The transcripts were combined into one dataset in NVivo and read in full by two researchers (T.P. (Tracey Price) and P.M.), then coded line by line. After coding the first four transcripts, the researchers met to discuss the initial framework and check analytic consistency. Finally, we introduced a deductive element, where the RTM framework and other relevant theories [46,47,48,49,50] were used to guide the development of analytic coding. The developing analytic framework was then used to code the remaining transcripts. H.C., P.M., T.P. (Tracey Price) and D.F. met regularly to discuss themes and their relationship to existing literature and the theoretical framework. Finally, the data were sorted into relevant themes and illustrative quotes chosen (by H.C. and T.P. (Tracey Price)).

### 2.3. Quantitative Data Collection and Analysis

All participants were asked to complete the Maslach Burnout Inventory (MBI; [51]). The MBI is a validated 22-item measure of occupational burnout, and respondents rate each item on a seven-point Likert scale, from ‘never’ to ‘everyday’. It views burnout as a continuum, from low to high, across three dimensions: emotional exhaustion (EE; feeling exhausted by one’s work), depersonalisation (DP; being emotionally detached), and personal accomplishment (PA; feelings of competence and success in one’s work) [51]. This study used the human services workers (MBI-HSS) version. To complete the measure, all participants were sent a link to the online survey. The responses were confidential: we did not ask for names or other identifiable information, so we are unable to match individual survey and interview responses. The MBI was used to triangulate the interview data and provide a greater understanding of participants’ experiences by showing whether the MBI scores reflected participants’ views and experiences. Informed consent for the MBI was granted by participation in the survey. Data from the completed MBI surveys were downloaded and scored using the MBI manual [52]. Each item was scored from 0 to 6 and then the scores for the items across the three sub-scales (EE, DP and PA) were added together, to provide sub-scale scores for each individual. These were then entered into Excel by D.F. and analysed using descriptive statistics. To interpret the scores, we used ‘cut-off scores’ [53]. Because Maslach and colleagues warn against putting too much stock in arbitrary ‘cut-off scores’ [54], we only used these cut-offs as a means of indicating general patterns in the data, where someone may be at higher risk of, or displaying indicators of, EE, DP or low PA. Each of the three sub-scores is interpreted separately, rather than being combined to form an overall burnout score. EE scores are interpreted as: low (0–16), moderate (17–26) and high (27+); DP scores are interpreted as: low (0–6), moderate (7–12) and high (13+); and PA scores are interpretated as: low (0–31), moderate (32–38) and high (39+) [53]. High EE and DP scores are indicative of burnout, whereas the opposite is true of PA, in which a low score indicates potential burnout.

## 3. Findings

A total of 18 individuals participated in an interview, and 11 also completed the MBI. Five participants were male, and 13 were female. Participants were from six organisations, with most working across central Scotland, and held various roles, including support worker/practitioner, housing support officer, nurse, keyworker, lead practitioner, and volunteer coordinator. The interview findings are presented first followed by the MBI findings.

### 3.1. Qualitative Findings

The data are organised into two thematic categories: firstly, pre-pandemic experiences, followed by experiences during the COVID-19 pandemic. Sub-themes within each category are described (using sub-headings). Pseudonyms have been used for each participant. Table 1 below details the themes and sub-themes.

#### 3.1.1. Pre-Pandemic Experiences

Participants described varying levels of stress experienced in their work prior to the pandemic. These experiences are seen as the ‘primary stressor’ in the RTM. Self-appraisal of stress was influenced by job demands, workplace culture, team dynamics, and communication. Participants discussed the demanding nature of their roles, working with people with complex needs as part of high caseloads.

##### Emotional Impact of the Role

Almost all participants had been drawn to their roles by a desire to make a difference. For some, this led to role conflict related to the complexity of the homelessness system, where it was not always possible to access accommodation or wider services for those in need. Empathy towards situations that clients were facing was viewed by many as being a part of providing a good standard of care, and this linked to the sense of making a difference. Some participants, however, experienced empathy as stressful, particularly where it had not been possible to access services for clients:

*Feeling frustration that you can’t get people access to services quick enough. That is also really difficult… Just it can be quite stressful, and quite upsetting.* (Sarah)

Several participants described how challenging they found it to explain to clients how the wider housing system worked and how frustrated it made them feel. Working in homelessness services itself was characterised as demanding by some participants. Wendy described the services as ‘chaotic’ and believed that the work done in homelessness services was viewed as insignificant by other sectors and professionals. Participants described not always having their knowledge valued when speaking with other professionals, which could reduce the support networks/resources available to them, a key element of social capital. Steve noted associated challenges of working in a sector that is ‘undervalued’, and the impact this can have on the staffing of services:

*[…] some social workers do look upon support work as a sort of cobbled together half, you know, half-way professional, an inadequate bunch of, you know, just carers almost.* (Steve)

Two participants told us that, over time, they began to emotionally distance themselves from clients to cope with the frustration involved in feeling empathy within situations that were out of their control. Control, or lack of control, over the ability to access services, and help clients to achieve their aims, appeared to link to perceptions of stress. Likewise, being perceived as *less than* other professionals also contributed to feelings of not being in control and of frustration.

Several participants described indicators of burnout when they recounted their experiences of work pre-pandemic. One participant in particular used dehumanising language to describe a traumatic incident and viewed not taking up the emotional support that was offered as a strength. Those who described having worked in the sector for a long time tended to talk more about professional boundaries and emotional distance as a coping strategy. Despite the many challenges experienced, several described using self-care and support to ensure that practice remained emotionally warm and empathetic, describing their roles as ‘fulfilling’.

##### Pre-Pandemic Workplace Culture

We asked participants several questions about their experiences of staffing, policies, and team working practices before COVID-19. We found varied experiences of workplace culture. Those who described culture in positive terms tended to discuss reflective cultures where it was acceptable to approach managers and admit to having a bad day. Staff valued managers’ practices of ‘cutting them some slack’ by relieving pressure and allowing staff to rearrange their diaries accordingly. As well as having approachable and supportive managers, many participants described a positive and supportive team culture in general which appeared to strengthen staff resilience and wellbeing. Those who described workplace culture in negative ways described the opposite: having to pretend to be okay and to demonstrate being excessively busy at all times.

Some participants described long-standing issues with high staff turnover in their service and having to take on additional tasks because of low staffing levels. Working in an understaffed service, alongside punitive absenteeism policies, resulted in people feeling that they had to come to work even if mentally or physically unwell, to avoid sanctions such as the threat of job loss and to keep the service going:

*Quite a lot of the time, staff come into their work when it’s quite obvious, whether that’s physical or mental health, that they shouldn’t be at their work, because they fear what will happen to them if they stay off. [Regarding the new absence policy] I think during COVID, you know, it could have waited. It didn’t have to be done during that period of time when staff nerves were tense and things were so uncertain. And we all had enough going on without that kind of hanging over you and knowing you were going in for that.* (Rebecca)

For some participants, this was experienced as having low control over how to manage stress, leading to frustration. Several participants described turning to other team members for support as an alternative to approaching management. In some cases, participants described a division between staff and management with a ‘them and us’ culture. In less supportive team environments, some participants described frustrations with colleagues. This related to a lack of shared values and norms regarding the approach taken within the services. The frustration commonly came from a disagreement regarding drug/alcohol harm reduction practices and a concern about management’s lack of response to undesirable or unethical practice within the staff team:

*It can be difficult to swallow it down sometimes though like sometimes I want to just scream at management, like you, like certain people shouldn’t fucking be here, if that is how they are conducting themselves, (a) how did they get the job in the first place, (b) how have they still got the job, and why is no one else freaking out?* (Chris)

##### Relationships with Clients

Participants generally talked about having good relationships with clients and building trust in spite of the challenges:

*It’s their own property so they feel more relaxed and you can sit there for an hour and a half and you just have this open conversation and then when they start building up the trust then they start telling you things about their life.* (Lynsey)

Relationships with clients were a motivator for many participants for doing the role. Some described time with clients as a welcome reprieve from workplace stress and something they experienced as fulfilling:

*So, the way I cope with it is I take myself out of the situation and I will spend a lot more time going out visiting my service users.* (Lynsey)

The findings presented in this section indicate that many participants were experiencing the working environment and demands of the role as stressful before the COVID-19 pandemic occurred. Positive, reflective cultures tended to increase staff resilience to the experience of stress, and many participants spoke about valuing the approachability of managers to discuss challenging situations. Findings also indicate that there was a pre-existing division between staff and managers in some services before the pandemic. This appeared to relate to non-reflective cultures, where struggling to cope was viewed as a weakness, and there was pressure to keep going regardless of the emotional impact. In some instances, this led to strong relationships between team members, but in others there was discord within staff teams related to a lack of agreement regarding an approach to issues such as alcohol or drugs and concerns about unethical practice.

#### 3.1.2. Experiences during the COVID-19 Pandemic

We now turn to participants’ experiences during the COVID-19 pandemic, the ‘secondary stressor’ in the RTM. As anticipated, the pandemic disrupted participants’ lives, creating a range of challenges in delivering services and to how staff were able to provide support to clients. For example, under the Scottish Government’s COVID-19 lockdown restrictions, visiting clients who had newly acquired tenancies was no longer allowed, and many participants expressed worry about the ability of clients to access basic essentials such as food. We first examine positive aspects before exploring organisational culture and coping strategies.

##### Positive Aspects of the Pandemic

Despite the challenges and changes that the pandemic brought, some participants identified positive outcomes. One reflected that she had very quickly found ways to maintain client contact and been supported to establish an assertive outreach programme where she would shop for clients with no informal support networks who were shielding (those deemed clinically vulnerable were asked to stay at home) in new tenancies. Although the visits were limited to doorstep interactions, the participant reflected that these were important to maintain social contact with clients and ensure that basic needs were met. Additionally, the Scottish pandemic response of converting vacant hotels into emergency homeless accommodation had further positive outcomes, as Margaret highlighted:

*We’ve been able to build really, really good relationships with people because we see them every day. And, you know, if we need something for them, we can just like go and knock on their door basically which has been so beneficial… with the hotels, and kind of bringing everyone into one space, we’ve been able to take more of the lead on that and so we’ve been able to really just build those relationships with people.* (Margaret)

Participants also described a greater focus on client involvement in how services were operating during the pandemic. Some participants discussed feeling empowered to provide feedback to their organisations and had participated in weekly online meetings with organisational directors. For some participants, being in direct contact with senior managers, and able to provide feedback, appeared to have led to increases in the number of support networks available to them (i.e., social capital). For some, this contributed to a sense of solidarity and a feeling of ‘belonging’ to the organisation. A few participants reflected that the shared experience of global ‘crisis’ had made people more aware of each other’s vulnerabilities and life circumstances and there was a sense of ‘we are all in this together’.

##### Working with Clients during the Pandemic

Despite the positive aspects of working during the pandemic, many participants talked about challenges related to new ways of working and ensuring continuity of support for clients. Some described feeling unable to control the risks posed by potential COVID-19 transmission. Low control over exposure to the virus (the secondary stressor) was described as stressful by many participants. As Chris and Wendy describe, trying to explain the seriousness of the pandemic to people who felt uncared for by society was very difficult:

*They feel like society doesn’t give a shit about them for the last twenty years. And now you want them to act as if they need to take responsibility and keep everyone else safe.* (Chris)

*It’s that kind of all the time “wash your hands, don’t touch this”… it’s like social distancing, you know, it’s constantly having to be on alert… especially for the client group, they are not very good at social distancing. They are not particularly good at, kind of, looking after themselves. So, it’s having to step up that.* (Wendy)

Participants described the early weeks of the pandemic as a time of uncertainty, where there had often been a lack of communication as to how to manage client care and support. Some described having to use intuition in the absence of information from managers or the organisation, having to make decisions about how to work best with clients, manage workloads and other considerations, when usually support would be provided. Some participants described the requirement of having to use PPE, such as face coverings and gloves, as a barrier to providing emotional support to clients:

*People are disclosing the most horrific things that have ever happened to them and, it does, it felt like a physical barrier between us, where if someone was to become upset, you know, in the right context you could tell the women was okay with that, you could go over and give them a cuddle or rub their back or, you know, appropriate touch, if you knew that the woman was okay with that. But you can’t do that because of COVID.* (Rebecca)

Others described how difficult it was to explain the need for barriers and PPE which seemed to unintentionally convey the exclusion that many of their clients had experienced throughout their lives:

*These are people who are used to exclusion. They have been excluded maybe throughout their whole life, or in parts of their lives, and now we have this kind of culture of exclusion because of COVID. And it’s not malicious, it’s just that it has to happen for health and safety, and it’s to protect people. But often we find now that they kind of put a barrier up when faced with that exclusion because it’s what they are used to in their past, and they maybe don’t understand that what we are doing is trying to protect them and keep them safe. And keep us safe as well so that we can come into work every day and keep doing what we are doing.* (Alex)

Some staff described going to great lengths to ensure relationships were maintained as much as possible. These changes meant that staff had extra time to spend with clients, were innovative in how they could respond to clients’ needs and were more consistent in their work with clients. At times, such adaptations required going against protocols to meet clients in person to ensure that clients’ needs and staff–client relationships were prioritised. Going against these protocols was described positively by participants.

##### The Impact of the Pandemic on Organisational Culture

The pandemic appeared to have acted as a magnifying glass for pre-existing positive and negative organisational cultures and practices. First, we describe positive aspects, followed by negative aspects of organisational culture.

While the pandemic caused a range of challenges for staff, those working in organisations with supportive and reflective cultures, strong team bonds, and strong relationships with managers described their experiences more positively. Being able to approach managers for support as and when needed was described as helping to offset experiences of stress:

*Management have sort of made themselves more open for like ad-hoc supervisions, I suppose like, you know, if you are struggling with something you can just call.* (Andy)

Participants described being able to open up about how they felt in relation to work, the pandemic, and the impact on their own wellbeing. In some settings, the pandemic had strengthened a focus on wellbeing and had further developed a culture where it was acceptable to admit to not being okay, and where there was an expectation that adaptations would be made to aid coping. Adaptations included being able to go home early, or juggle appointments, as necessary. This flexibility and the ability to be honest about coping led to a feeling of trust, where vulnerability was normalised and accepted. Often, this feeling of solidarity and shared human experience was extended to clients, and participants described being open with clients about their own emotions, leading to more reciprocity within relationships, and increased bonding between staff and clients. Ad hoc, responsive and non-judgmental check-ins from supportive managers helped to maintain a positive reflective culture, where people pulled together to navigate what emerged as a very human crisis:

*Vulnerabilities, people acted, people act differently. It’s funny, people were, you know, you see how the team, you know, I’m going to be the one who is going to do this, I’m going to be the one who will do that, right can you, and everything has kind of, I don’t know how it all fell into place but it just did. It just, everything worked… I think when people are scared, then you see the real people.* (Wendy)

Where managers encouraged this, the organisational culture was experienced as embracing reflection and emotional honesty. This then produced a sense of gratitude and worth, key elements of social capital, which was extended to clients in a very human sense of bonding through crisis and adapting together. The sense of ‘all being in this together’ was experienced in well-functioning teams. We refer to this as solidarity and note that it seemed to be a contributing factor that supported coping and resilience among those interviewed.

On the other hand, some participants talked about the negative impact of the pandemic on organisations which were more hierarchical in their organisational form, and where there were clearer divisions between staff and management. According to participants, in these situations, the pandemic appeared to exacerbate pre-existing tension and strain, leading to a sense of further fragmentation between staff teams and managers and, between staff and clients:

*The problems that we had became magnified. I am a great believer in when people go into crisis, organisations go into crisis. They don’t tend to change, they tend to do more of what they do, and so the communication became an issue, kind of hierarchical, and people were, you know, understandably, everybody was scared. There was fear, and at the same time we were trying to deal with the clients, and for periods we were kind of mirroring their fear and uncertainty, and they were mirroring our fear and uncertainty. There were lots of decisions which sometimes led to a bit of chaotic practice.* (Mike)

Frustration was commonly reported around organisational policies, a lack of guidance about COVID-19 regulations, and a lack of support to guide practice changes. Some participants stated that policies should have been introduced to facilitate navigation of the changes brought about by the pandemic, and a lack of official guidance was a source of dissatisfaction. Where there was a pre-existing ‘them and us’ culture, this was experienced as exacerbating tensions, particularly because staff who remained on ‘the frontline’ believed that they were not consulted on the implications of this:

*I just felt it was like “so you all get on with it, but we will not be here. We are not going to be in the office”. So, I just think the support just disappeared. I felt as if it was like right, we are going to look after ourselves… A lot of the staff were really scared about what do we do?* (Lynsey)

*The people who get paid the least, they are the people that are, like, working the hardest and working, you know, night shifts, and doing sleepover shifts. And they are the ones that are also putting their health and safety on the line.* (Margaret)

A sense of shared frustration about managers, and feeling a lack of control and autonomy, appeared to act as glue to unify some teams, creating stronger team bonds. However, this lack of autonomy seemed to create further separation between management and staff, resulting in closed groups. Some participants described feeling as though managers did not know what was happening within the service settings.

##### Reflection, Supervision and Training

Participants discussed particular aspects of organisational culture as mediating their experiences of stress during the pandemic. Aspects such as reflective practice opportunities, supervision, training, and the overall organisation ethos were viewed positively by most participants:

*So, like, if something bad happened I was just like, I’d speak to her [Manager] and debrief and stuff, and she was like really supportive of me and then she was always reassuring me that I’d done my best.* (Teresa)

Informal peer support, supervision, and reflective practice sessions between staff and within staff teams existed, as well as more formal supervision with line managers. For several participants, reflective practice was delivered by an external person, often a Clinical Psychologist. Some settings provided a range of opportunities for formal reflective practice:

*[Organisation] do… reflective practice every week, where one of the local psychiatrists, psychologists… they do every week there was a chance and then there was a big one every month.* (Colin)

Two participants talked about the potential downsides of reflective practice and supervision sessions. One noted that such sessions could increase stress in circumstances where there was insufficient time allocated within their workload. A few participants also described reflective meetings that took place in organisations where the culture did not support reflecting openly on emotions. In such settings, reflective meetings were described as ‘tokenistic’:

*It [reflective meeting] hasn’t happened for a wee while. It happened a couple of times and there was a lack of structure, there was a lack of information about what reflective practice could be or should be, or whatever […] We are very hierarchical, top down, and there is a lot of blame flies around in this service. Sometimes it’s deserved, you know, everybody makes mistakes, but as a culture that inhibits discussion.* (Rebecca)

Another pointed out that communication concerning the purpose of reflective meetings had been unclear, and stated that the meetings had become somewhere to discuss problems, rather than solutions, which could exacerbate staff frustrations:

*If the [Organisation] had maybe done one for a longer length of time, then that frustration and anger wouldn’t have been there. Because they maybe would have known this is what reflective practice is for. It’s to sit down and look at issues or aspects or something that has happened and, you know, could it be done better, and could we have worked in a different way? It became a blame game, you know? “This is your fault this happened”, and that’s not the culture you want.* (Colin)

Participants who described positive reflective cultures in their workplaces tended to value ad hoc, informal reflection via impromptu discussions with managers:

*There is always that opportunity where it’s a real, it’s a check in, you know? Where things are at. And they are always asking if there is any, you know, any issues with the clients, the team, and you do talk through each of your caseloads, for that reason. So, there is that stuff that is in place. As I say, there is the impromptu kind of stuff where you can just say “look I’m really struggling with this person” or, I need to just, or you just come in and debrief them what has happened.* (Wendy)

Because of the COVID-19 restrictions, some supervision and reflective practice sessions had been provided online, rather than face to face. While participants were appreciative of such sessions, there was a dislike of the online format. Many described finding it difficult to engage, as described by Andy:

*I’ve hated it. I hate being on a webcam […] I certainly miss the face-to-face contact and being able to sit in a, you know, in an office and even just have a general chat. It might not be about work but just having that contact with the team, I certainly missed that. And I know quite a lot of the team were feeling the same, you know, they have said that over Zoom, you know, that they can’t wait for the face-to-face meetings to resume. But I certainly find it helpful though. I think it’s better than not, not having any contact.* (Andy)

Participants much preferred face-to-face sessions and talked positively of the opportunities available during the summer of 2020, when restrictions eased and they could meet outside with colleagues.

##### Coping Strategies

We asked participants about the strategies they had used to help cope with the stress of the role prior to the COVID-19 pandemic, and to reflect on how these might have changed during the pandemic. Both before and during the pandemic, many participants expressed that they found it difficult to switch off from work. Several participants described using meditation, mindfulness, and breathing exercises to calm intrusive thoughts. Others told us that walking outside and being in nature helped to reduce feelings of anxiety. Several participants expressed that spending time with their pets, either at home or while outside, helped them to cope:

*We recently rescued a cat and like my cat is my best therapist.* (Chris)

For many, physical exercise had been an important coping mechanism before the pandemic. The COVID-19 restrictions had meant that gyms were closed and participants described adapting to this by cycling or swimming outdoors as part of a suite of new coping strategies. Several participants were acutely aware of the strategies needed to support their own wellbeing, and saw regular breaks outside as important elements of self-care:

*I have a policy of getting out of the building as often as I can. That’s my, I have always had that, we get two half-hour breaks in our shifts and I’m quite renowned for making sure I get out. I’ve always done that, so I’ve carried on with that.* (Mike)

Some participants expressed that social contact with friends and family had helped with coping pre-pandemic and were acutely feeling the loss of social contact that had occurred as a result of COVID-19 restrictions. This tended to be particularly significant to those who were living alone. For these participants, time with clients and colleagues was an important source of social contact during the lockdown periods, at a time when only essential social contact was permitted. Many described using online platforms to stay in contact with family and friends. Several participants expressed that the support of household members was important to aid coping during the pandemic:

*I’m just in a really fortunate position where I’ve got a really supportive husband, I’ve got a really supportive family and, like I said, I have got great managers and colleagues. I think if I didn’t have that then yeah, this would be very different.* (Sarah)

Some workplaces were not permitting annual leave during the pandemic, and many participants described feeling exhausted and in need of a break. Steve talked at length about the challenges of annual leave during the pandemic and the impact this had on team members. For him, taking time off was an important way of dealing with stress. Relatedly, in other settings where annual leave had been permitted, some participants reported that they and other colleagues had taken decisions not to use annual leave because restrictions meant that travel plans were cancelled. Although these decisions had been taken voluntarily, many participants reflected that they were feeling exhausted and in need of time off to recuperate:

*I wouldn’t say it’s been high stress but emotional exhaustion definitely… just feeling a bit I need a holiday, but I can’t go anywhere… But yeah, I am thinking of taking some annual leave, even just to stay at home and, you know, keep the laptop and phone away.* (Andy)

Some participants felt that their coping abilities were beginning to reduce as a result of exhaustion:

*I felt like my level of patience was… like my fuse was a lot shorter. So, I definitely had to work harder at work to be more present […] which definitely made me exhausted. So, it was sort of just a big circle of emotions.* (Christina)

Some described operating on adrenaline at the start of the pandemic, feeling energised by the work, and able to deal with the fast pace of change. For several, this additional energy began to diminish over time:

*The first few months it almost was yes, I don’t even know how to describe it, it was just great, like I honestly just wasn’t tired. I was like surprised at myself how energised I was. I literally like jumped out of bed every morning and was ready to go for the day. And then it just seemed to be at one point it just… came crashing down a little […] I think yes just a little bit deflated, and I think I was yes, just definitely, emotionally exhausted is definitely how I would describe it.* (Margaret)

Participants talked about other ways of helping them cope with the stress and anxiety experienced as a result of the pandemic, including access to counselling, support from management and colleagues to maintain their usual coping strategies, and encouragement to take annual leave. Despite many positive examples of coping strategies, some participants described avoidant styles of coping, such as keeping busy to avoid thinking or feeling, and using alcohol after work as a way to mitigate stress:

*I tend to find myself in ever repeating patterns of certain behaviours… Like I will address it and it will become unmanageable, or it will become less damaging for a period of time, and then something else will happen, or I will take my eye off the ball, or I will not be doing the physiological self-care stuff. And I will let something slip and rather than go to the gym, I will do something else that is not as good for me that is easier to do, you know, the lazy option, the quick fix.* (Chris)

One participant described frustration becoming dislocated and manifesting in other ways:

*This morning I could have put this PC though the window because it was winding me up so much […] I tear my hair out with IT stuff, then not, the actual, the challenging aspects of support work and homelessness work and addictions work doesn’t stress me out … but that’s maybe that’s just stress in general manifesting itself in me getting wound up by this computer.* (Steve)

##### Recommendations for the Future

When reflecting on the lessons that could be learned from their experiences of frontline working during the pandemic, participants considered the recommendations that could be made. Several participants suggested that improvements should be made to pay and conditions within the sector, suggesting that this would help staff to feel more valued and appreciated. Many participants emphasised annual leave as fundamentally important to wellbeing and felt that there should have been policies to ensure that people were taking time off when entitled to do so. In a few circumstances, participants recounted that annual leave had not been permitted during the pandemic and recommended that this should not occur again in the future, due to the detrimental effect on staff wellbeing. Most participants also felt that staff wellbeing should be prioritised:

*I’ve always struggled to understand why staff wellbeing… is an add on. It’s not like a core function.* (Mike)

For some, supervision played a key role in maintaining wellbeing and ensuring a supportive organisational culture. Those who felt this way emphasised that their positive experiences were not universal across the sector, and that more should be done to ensure positive organisational cultures where staff wellbeing was seen as a priority. Other participants recommended that counselling and/or therapeutic services be made more readily available to staff. For several, this would contribute to feeling valued, and would also help to support coping skills:

*Maybe some extra access to like maybe counselling services would have been appropriate, because I’d say a lot of people in my team have been struggling with depression and anxiety.* (Andy)

In one example, the offer of counselling for vicarious trauma had been made but not taken up, though the offer had been experienced as supportive. In one setting, staff had been given two wellbeing days that could be taken at short notice, which had been experienced as supportive, and something that one participant was keen to recommend.

In summary, participants recommended that organisational practices should ensure that staff feel valued and that their wellbeing is prioritised. Participants recommended that annual leave be actively encouraged, supervisory practices be strengthened, and access to therapeutic services increased. Participants also recommended that punitive absence policies should be not have been implemented at a time of stress and in future should be more understanding towards people’s circumstances.

### 3.2. Quantitative Findings

Overall, participants showed low/moderate levels of emotional exhaustion (EE), low depersonalisation (DP), and moderate levels of personal accomplishment (PA), as measured by the MBI. Table 2 details the mean, median, and range for each dimension. When examining the scores, it is important to note that, whilst high EE and DP scores are indicative of burnout, the opposite is true of PA, in which a low score indicates potential burnout.

When examining the scores by each individual dimension, we found that, while most participants had low EE scores (i.e., 0–16), 3/11 had moderate scores (17–26), and 2/11 had high scores (27+). The median score of 18 for all participants is suggestive of moderate levels of emotional exhaustion, indicating that approximately half the sample (5/11) were either experiencing relatively high levels of emotional exhaustion, or were at ‘risk’ (i.e., experiencing ‘moderate’ levels). For DP, the mean and median scores suggest low levels of depersonalisation, while 2/11 had scores indicating moderate levels (7–12), and another 2/11 reported high levels (13+). For PA, the mean and median scores are suggestive of moderate levels of personal accomplishment (32–38), with 1/11 scoring very high (39+). Overall, while most scores suggested participants were not experiencing burnout, 2/11 scored at higher levels. The findings indicate that, at the time of data collection, participants were not experiencing burnout but may be at risk in the future if issues were left unaddressed.

## 4. Discussion

This mixed-methods study explored experiences of stress and wellbeing of third sector (not-for-profit) homelessness services workers during the early stages of the COVID-19 pandemic in Scotland. It is the first UK study to explore the views of those working on the frontline in homelessness services during the pandemic. As mentioned previously, a great deal of research focused on the experiences of frontline health care workers during the pandemic, with very little being focused on those working in homelessness services (e.g., [32]). Our findings suggest that the COVID-19 pandemic greatly impacted those interviewed and seemed to magnify both the positive and negative aspects of the frontline homelessness service role and organisational culture, within several homelessness services in Scotland. Prior to the pandemic, participants described their roles as demanding and involving having to work in challenging situations, as well as having good relationships with clients. They also described varying aspects of organisational culture. Previous research has also highlighted similar themes from those working within the homelessness sector (e.g., [4,5,10,17]). Our participants described situations where teams who were functioning well before the pandemic seemed to work effectively together during the time of crisis, and bonds were further strengthened. Participants noted that this tended to occur in organisational cultures where reflexivity was embraced and strengthened by managers who were aware of the emotional needs of staff members, regularly checked in to ask how things were going, and proactively identified situations where support was required. Conversely, other participants described fragmentation between managers and staff before the COVID-19 pandemic, which they noted resulted in stress and challenging working environments. Participants in these services described feeling angry when, at the start of the pandemic, managers were able to work from home and staff remained on the frontline. Participants discussed these tensions in relation to a lack of communication that exacerbated a pre-existing ‘them and us’ culture between staff members and management, whilst appearing to strengthen relationships within staff teams. In some settings, formal reflective practice was provided but was experienced as tokenistic. In these settings, staff reported feeling unable to be honest about their emotions, their coping abilities, or any challenges faced within their roles. Some participants described taking breaks during the working day or annual leave as essential to coping with working during the pandemic, while others discussed punitive responses to absenteeism and not being allowed to take holidays.

Participants’ descriptions of organisational culture during the pandemic is reminiscent of the ‘blitz spirit’ described by Furedi (2007) who noted that the sense of potential impending disaster during the London bombings of World War II were mitigated by a sense of solidarity, togetherness, and shared experience [55]. Our findings reflect this spirit of togetherness during the COVID-19 pandemic but illustrate that such feelings of solidarity were strongest among those who felt well supported by managers and able to freely express their feelings of vulnerability and any concerns. This finding is consistent with wider natural disaster literature where solidarity boosts resilience of populations and groups through a shared sense of reciprocal care, and a willingness to make sacrifices to promote the wellbeing of others [47,48].

Our quantitative findings are consistent with the qualitative findings. Although interviewed participants described coping generally, they identified a number of issues and challenges indicating potential risks for burnout in the longer term. Levels of personal accomplishment (PA), depersonalisation (DP), and emotional exhaustion (EE) indicative of ‘burnout’ develop over time. Our data indicate that several participants may have been at higher risk of burnout when data were collected, due to their moderate/high EE/DP scores, and low/moderate PA scores. The low levels of depersonalisation were also indicative of the qualitative findings. Typically, participants spoke about empathy and connection with their clients, in person-centred and rights-based language, albeit to varying degrees. There were a couple of instances of depersonalised, emotionally colder and distanced language, indicative of higher depersonalisation scores. The lower levels of personal accomplishment are representative of the general frustration which participants discussed in the interviews. They spoke about both COVID-19 specific barriers to providing care and helping clients progress, as well as concerns about more general organisational constraints and structural issues, such as a lack of available housing or non-trauma-informed external organisations. These frustrations are consistent with the lower levels of personal accomplishment in the Maslach Burnout Inventory (MBI), as participants felt there were several barriers constraining their capacity to make a difference. It is also important to note that, while studies examining burnout using the MBI in homelessness services are scarce, our findings show similarities with the limited evidence base (e.g., [56,57,58]).

Drawing on the Goh et al. (2010) Revised Transactional Model (RTM) [42], our study provides an understanding of the ways in which stressful events were experienced by participants. In the RTM, people’s stress levels will be impacted by their coping strategies and available resources when they are faced with stressful events. In this study, pre-pandemic working conditions (the primary stressor), and individuals’ available coping strategies, influenced their experiences of work during the pandemic (secondary stressor). Our findings have added the influence of control, social capital and solidarity to the model, which acted as additional resources for our participants during a challenging time. Our findings indicate that someone’s perceptions of their control over the stressor (i.e., levels of autonomy, control over clients’ situations, and also over the impact of COVID-19) was influenced by the level of social capital they perceive they have (i.e., their support networks and resources). High social capital (i.e., good social networks) was found to come from approachable and adaptable managers who listened and responded to staff perceptions of their needs, as well as the needs of clients and the service. This seemed to result in feelings of control over the secondary stressor (the pandemic) which offset enough stress to make the potential primary stressor (pre-pandemic organisational context) feel less risky to staff wellbeing. Participants talked about how having control and a sense of solidarity in their teams facilitated feelings of being able to cope with the challenges experienced. Others described situations where they did not have these high levels of social capital, and therefore did not feel in control and the pandemic caused them to feel additional stress. These negative experiences appeared to be compounded by difficult working environments and unsupportive management.

While the pandemic intensified social bonds and solidarity in some cases, it also appeared to magnify the polarisation that occurred in others. This suggests that, while this study provides an important contribution to understanding staff wellbeing at a time of global crisis, it also provides an insight into coping more generally. Notably, interviews were conducted several months into the pandemic (June–October 2020). At this point, participants self-identified as being physically exhausted, with many indicating that they felt in need of time off to rest. Participants described a range of recommendations relating to organisational culture, such as improvements to pay and conditions, opportunities to take annual leave, and access to supervision and counselling. Previous studies have also highlighted the influence of organisational culture and the wider homelessness system on staff wellbeing [5,9,19]. This study adds to the evidence base by providing an insight into the additional challenges (as well as perceived positives) stemming from working in homelessness services in the UK during a global pandemic.

### 4.1. Implications for Policy, Practice and Research

In terms of policy and practice, our findings highlight the need for clear support structures for those working in frontline homelessness service roles, with relevance to the UK and beyond. Participants described stressors such as lone working, high workloads, and high levels of responsibility, whilst navigating a complex system, which have also been described in international studies. Although many of the challenges described by participants were not specific to the pandemic, they appeared to be exacerbated by it, suggesting a need to address concerns and difficulties post-pandemic. Service managers should be supported to work closely with staff to ensure clear communication and encourage a culture of flexibility and autonomy. Additionally, services should be encouraged and supported to develop a sense of solidarity between service managers, staff and clients. This can be supported by reflective cultures, team building, and good communication, all of which were highlighted by participants. Relatedly, services should provide opportunities for informal and formal communication with staff, to ensure staff feel listened to and supported. Fourthly, reflective practice appears to be important at an organisational level and can be embedded into organisational cultures. This includes reflective supervision, which should be provided by well-trained professionals who are external to the staff team and management, where possible. Some participants spoke highly of their organisation’s reflective practice culture, whereas others described this as tokenistic. Embedding person-centred reflective practice at the organisation and sector levels could ensure staff are well supported in their roles. Finally, staff discussed the importance of time off. Staff should be protected from exhaustion, through encouraging (allowing) them to take annual leave and providing access to counselling and other supports. Given the policy focus on addressing homelessness in Scotland, as well as within the UK and internationally, there should be emphasis on supporting staff at the sector level, to ensure equity across organisations.

In terms of implications for research, it is important to note that this study was conducted in the summer of 2020, in the early phases of the pandemic, and therefore only provides a snapshot of participants’ experiences and within a particular time and place. Given the ongoing nature of the COVID-19 pandemic, and the likelihood of burnout developing over a longer period, further research is required to understand the longer-term impact of the pandemic on the wellbeing of frontline staff working in homelessness services, and to explore changes over time. It would also be beneficial for future studies to examine in more depth the key themes raised in this study, such as the impact of reflective practice, good communication within teams, solidarity, and protection from exhaustion, on staff outcomes. It would also be beneficial to administer the MBI with a larger population of participants to assess levels of burnout in the sector more widely. Given the lack of research into the experiences of those working in homelessness services, future research in this field is essential.

### 4.2. Strengths and Limitations

This study provides insight into experiences of stress and wellbeing of frontline homelessness workers in Scotland during the COVID-19 pandemic. Despite the clear challenges that organisations and their staff were experiencing, we were able to interview 18 individuals who worked in a variety of roles in six different homelessness organisations across Scotland. The diversity of participants allowed us to capture a breadth of different experiences, providing a good understanding of work during the pandemic. The use of the MBI enabled us to capture data on different elements of burnout and triangulate our qualitative data. Using the RTM to inform our data collection and analysis also allowed for a greater understanding of the potential key factors influencing participants’ experiences of stress and wellbeing.

It is important to note that, while our participants’ experiences were varied, it is likely that those who were already experiencing high levels of burnout did not participate, due to feeling unable to participate in an additional work-related task, or because managers did not identify them for the research, or because they were absent from work. Our recruitment approach, of involving service managers as gatekeepers, may also have limited participants to those who were not yet experiencing burnout, and/or those who had more positive experiences. In some cases, managers passed on individual’s contact details, and in others, participants emailed the study team directly. However, it was unclear as to whether they had been asked to do so by managers or had chosen to do so of their own accord. While it would have been beneficial to directly approach staff without involving service managers, generally their contact details were not in the public domain. This was the reason why we utilised the approach of asking service managers to make initial contact. Finally, only 11 of the 18 participants completed the MBI, which means that our findings do not relate to the whole sample. Participants may have felt that the MBI was too intrusive, or that they did not have time to complete it, or simply forgot to do so. Despite this, the small sample still shows variation in terms of scores and experiences, which corroborate our qualitative findings. Due to the very small sample, we are not able to draw any further conclusions from these data.

## 5. Conclusions

This study provided insight into the experiences of stress and wellbeing, and other related factors, of frontline homelessness service staff during the early stages of the COVID-19 pandemic in Scotland. The findings highlighted the positive and negative experiences prior to the pandemic, which were exacerbated by the global crisis. For some, the pandemic strengthened team bonds and enabled staff to provide clients with greater support. For others, the pandemic led to additional frustrations, poor communication, and fragmented teams, which appeared to have a negative impact on wellbeing. Participants identified several factors that should be prioritised, both in their organisations and across the homelessness sector in Scotland, to support staff wellbeing. These included reflective practice cultures, access to counselling/support, open and honest team communication (particularly from management to staff), and ensuring staff are protected from exhaustion through ensuring they are able to take their annual leave. These recommendations are also likely relevant to other countries, given the similarities described in the literature from other countries. Future research should explore the longer-term impact of the pandemic on homelessness service staff outcomes and explore the most effective ways of providing support to try to mitigate staff stress and burnout.

## Figures and Tables

**Table 1:** Themes and sub-themes.

Pre-Pandemic Experiences	Experiences during the COVID-19 Pandemic
Emotional impact of the role	Positive aspects of the pandemic
Pre-pandemic workplace culture	Working with clients during the pandemic
Relationships with clients	The impact of the pandemic on organisational culture
	Reflection, supervision and training
	Coping strategies
	Recommendations for the future

**Table 2:** Maslach Burnout Inventory (MBI) scores for participants (*n* = 11).

Dimension	Mean	Median	Range
Emotional exhaustion (EE)	16.9	18	3–31
Depersonalisation (DP)	4.4	2	0–11
Personal accomplishment (PA)	34	32	26–46

## Data Availability

The datasets generated and/or analysed during this study are not publicly available. Individual privacy could be compromised if the dataset is shared due to the small sample involved.

## Supplementary Materials

The following supporting information can be downloaded at: https://www.mdpi.com/article/10.3390/ijerph19063659/s1, Supplementary File S1: Interview schedule.
