# Supplementary material for: Stress and Wellbeing during the COVID-19 Pandemic: A Mixed-Methods Exploration of Frontline Homelessness Services Staff Experiences in Scotland
Source: Int J Environ Res Public Health. 2022 Mar 19;19(6):3659. doi: 10.3390/ijerph19063659 (PMC8953212; doi:10.3390/ijerph19063659)
Supplement: Supplementary file 1 [file ijerph-19-03659-s001.zip › ijerph-1591123-supplementary.pdf]

Carver et al. Supplementary File S1
Interview schedule

1. Please can you start by telling me about your role?
   Probes: How long have you been in post? Who are the people you support? What does the staff team look like? What do you enjoy about the role? How would you describe the organisational culture?

2. What drew you toward this line of work?

3. How would you describe your relationships with your clients?
   Probes: Do you get on with everyone? Why/why not? Do you find it difficult to 'connect' with any of them? Why/why not?

4. Thinking about the 6 months before COVID-19 (i.e. between October 2019 and March 2020), how have you found working in the service?
   Probes: What has been good/bad/ok? Have you encountered any issues? How have these been dealt with? How have your relationships with colleagues/team dynamics been?

5. What are the most challenging aspects of your role, in general (not relating to COVID-19)?
   Probes: Why do you think these issues occur? What makes these things challenging?

6. Again, thinking about the 6 months before COVID-19 (i.e. between October 2019 and March 2020), have you experienced feelings of exhaustion as a result of your work or have you felt the need to distance yourself emotionally from your work, to help you cope?

   Probes: Can you identify what may have caused these experiences? Have you received any support (from your organisation or other) to address these problems? Have you felt less able than usual to engage with people (staff/clients)? Has the organisational culture allowed you to do this?

7. We know that stress can be improved when staff have access to reflective practice, supervision, staff meetings and training. Thinking about the 6 months before COVID-19 (i.e. between October 2019 and March 2020), have you been provided with the opportunity to take up these activities, or other related activities?
   Probes: What activities? How did you find them? Did you feel supported to access these activities? If no, would you have liked this opportunity? What would you have liked to have received?

8. Now thinking to the current COVID-19 pandemic (starting in March 2020), how have you found working in the service?
   Probes: What has been good/bad? Have you encountered any issues? How have these been dealt with? What has changed as a result of the pandemic? Has the organisational culture changed? Have relationships with colleagues/team dynamics changed?

9. Have you experienced stress, emotional exhaustion or related problems as a result of your work during the pandemic?
   Probes: Can you identify what may have caused these experiences? Have you received any support (from your organisation or other) to address these problems?

10. During this time, have you been provided with the opportunity to take up these activities (like supervision, reflective practice, staff meetings, training), or other related activities?
    Probes: What activities? How did you find them? Did you feel supported to access these activities? If not, what activities would you want to receive?

11. Have you been using any another other strategies to manage your stress levels at work as a result of the COVID-19 pandemic?
    Probes: These strategies could involve peer support; informal/formal counselling; self-care; exercise; mindfulness; medication etc. What strategies have you tried? What have you found effective/less effective and why?

12. Is there any other support you think you require as a result of working during the COVID-19 pandemic that you've not received?
    Probes: What kind of support? Why might you not have been able to receive it?

13. Thinking about the future, once lockdown has ended, what changes do you think may occur or need to occur in your workplace?
    Probes: Good/bad changes? What do you think of these? Changes to service, staff, and/or for clients?

14. What ongoing support/activities do you think you will need as the pandemic continues and afterwards?
    Probes: How might these activities help you and/or your colleagues?

15. Overall, what lessons can be learned in terms of staff wellbeing, stress and emotional wellbeing from your experiences? Is there more that could and should be done?
    Probes: What kinds of things? What changes might these make to how you work?

16. Is there anything else you would like to add?
